# Trapped in the process: procedural justice and emotional escalation in digital citizen-state interactions

**DOI:** 10.3389/fpsyg.2026.1844065

**Published:** 2026-07-29

**Authors:** Zhuoyuan Tang, Xiyang Yin, Ping Li, Xuan Pan

**Affiliations:** 1 School of Information Technology, Zhejiang Financial College, Hangzhou, China; 2 Faculty of Applied Sciences, Macao Polytechnic University, Macau, Macao SAR, China; 3 School of Law, Beijing Institute of Technology (Zhuhai), Zhuhai, China; 4 Medical Record, Zhejiang University School of Medicine Sir Run Run Shaw Hospital, Hangzhou, China

**Keywords:** aggregated emotional patterns, cognitive appraisal, digital citizen-state interactions, emotional escalation, perceived control, procedural justice, social cognition

## Abstract

Digital complaint systems generate large-scale traces of citizens' emotional expression, offering new opportunities for computational social psychology to examine how institutional settings shape collective appraisal patterns. Emotional intensity in these systems varies dramatically across governance domains, yet the psychological processes associated with this variation remain underexplored. Drawing on appraisal theory and procedural justice literature, we examine emotional intensity as a text-based proxy for aggregate appraisal tendencies related to procedural fairness, such as citizens' sense of control and perceived institutional responsiveness. Analyzing 76,447 submissions from a provincial digital governance platform in China, we combine BERTopic modeling with multi-dimensional sentiment analysis to map emotion-topic coupling patterns. Results show a clear gradient: domains tied to long-term welfare, such as labor rights and education, show the mildest emotional profiles, while housing disputes and consumer complaints show markedly stronger intensity. We highlight two structural patterns associated with this asymmetry. First, spatial-functional misalignment reveals a mismatch between citizens' holistic representations of localized problems and fragmented administrative processing. Second, linguistic markers in extreme-intensity submissions heavily emphasize procedural language (e.g., “rectification,” “transfer”) over crisis vocabulary, suggesting frustration with stalled pathways. We conceptualize this pattern as procedural entrapment: a form of emotional escalation associated with institutional fragmentation, diminished perceived control, and diffuse accountability. Beyond administrative efficiency, these findings suggest that emotional data can serve as diagnostic signals of institutional bottlenecks. The study also speaks to the United Nations' Sustainable Development Goal 16 (Peace, Justice and Strong Institutions) by examining how institutional design relates to perceived procedural justice and institutional legitimacy in digitally mediated citizen-state interactions.

## 1 Introduction

Culture provides interpretive resources through which people make sense of institutional encounters, including expectations about authority, responsibility, and responsiveness. Functioning as a publicly accessible system of symbols and norms (Patterson, 2014), this “public culture” becomes internalized into what Lizardo (2017) calls “personal culture,” shaping how individuals appraise state-citizen interactions. These internalized cultural dispositions operate within what Cerulo et al. (2021) describe as the interdependence of individual cognition and group-level meaning systems. In the Chinese context, for instance, traditional political culture is characterized by a hierarchical orientation toward authority in which government leaders are expected to bear broad responsibility for citizens' welfare (Shi, 2015). This orientation may foster relatively undifferentiated expectations of governmental problem-solving, wherein citizens approach complaint platforms with integrated representations of their problems rather than pre-sorting demands by functional jurisdiction. This gap between integrated citizen-facing expectations and segmented institutional routines offers a psychologically meaningful backdrop for examining why some complaint domains are associated with stronger emotional escalation than others.

While digital complaint systems are often studied as tools for administrative efficiency (Sharmin and Chowdhury, 2025), they also function as socio-psychological environments where citizens form appraisals of institutional fairness. In China, institutionalized digital feedback mechanisms now handle hundreds of thousands of submissions annually, linking grassroots demands directly to administrative departments and triggering concrete governmental responses even in centralized structures (Wang et al., 2025; Indiahono, 2021), with participation patterns varying sharply across regions and topics (Shen et al., 2024). The intensity of emotional expression in these systems varies dramatically across governance domains. While individual differences in traits such as emotional stability or resilience may shape personal thresholds for frustration, the systematic variation observed at the aggregate level suggests that structural features of the administrative environment also play an important role. Yet, the psychological processes associated with this macro-level variation remain underexplored. While these differences are often attributed to issue urgency, psychological research suggests a complementary perspective: individuals evaluate institutional interactions not only by material outcomes but also through their subjective experience of the procedure. According to procedural justice literature, individuals respond strongly to how fair, transparent, and predictable decision pathways appear, even when outcomes remain constant (Lind and Tyler, 1988; Tyler, 2006). When procedures feel opaque or evasive, strong discrete emotions—such as anger, frustration, and resentment—can emerge as reactions to perceived procedural injustice rather than the substantive issue alone (Weiss et al., 1999; Barclay et al., 2005). The interplay between procedural conditions, outcome favorability, and discrete emotional responses has been documented in organizational settings (Krehbiel and Cropanzano, 2000; Folger and Cropanzano, 1998), while research on interactional justice suggests that perceived disregard in interpersonal encounters can intensify negative reactions even when formal procedures appear adequate (Bies, 2001).

Computational text mining has enabled researchers to identify thematic structures and sentiment patterns in large-scale complaint data (Grootendorst, 2022), reflecting the broader turn toward computational social psychology as a method for examining psychological processes at population scale (Cushman, 2024). However, topic modeling and sentiment analysis typically operate as separate analytical tracks, identifying what citizens discuss and whether they express negative sentiment, but not why certain domains generate sharper emotional escalation. It remains unexplained, for instance, why housing disputes or consumer complaints consistently trigger stronger emotional reactions than pension enrollment or school admissions, although the latter concern citizens' long-term welfare.

A psychological perspective helps clarify this pattern. Research indicates that low perceived control generally amplifies negative affective responses (Skinner, 1996; Spector, 1986; Zhao et al., 2021), and classic motivation theory emphasizes individuals' intrinsic need to sustain a sense of effective agency (White, 1959). Appraisal theory further posits that the intensity of emotion reflects cognitive evaluations of relevance, goal-congruence, and controllability, rather than objective severity alone (Lazarus, 1991; Ellsworth and Scherer, 2003). From this standpoint, strong negative expressions in digital complaint systems may signal collective perceptions of blocked agency, ambiguous accountability, or circular referrals.

We advance procedural entrapment as an interpretive framework for this configuration. While prior research recognizes the negative impacts of administrative burden (Halling and Baekgaard, 2024) and learned helplessness—where individuals passively withdraw because they believe outcomes are independent of their actions (Seligman, 1972; Abramson et al., 1978; Maier and Seligman, 1976)—procedural entrapment describes a different dynamic. We use the term to describe complaint contexts in which formally available pathways require continuous citizen engagement yet appear difficult to navigate toward resolution. In appraisal-theoretic terms, this configuration is characterized by high perceived relevance, high goal incongruence, and—critically—ambiguous controllability: the pathway is formally open, yet perceived agency over resolution remains low (Lazarus, 1991; Ellsworth and Scherer, 2003). Unlike learned helplessness, where controllability is appraised as entirely absent and withdrawal follows, procedural entrapment sustains engagement while undermining the sense that engagement is effective. Repeated referrals or diffuse accountability may thus be associated with an active exhaustion of psychological resources and escalating negative affect. Emotional intensity may therefore serve as an aggregate, observable indicator of diminished perceived control.

By conceptualizing emotional intensity as an aggregate psychological response, this study explores how institutional structural characteristics correspond with citizens' emotional appraisals. Rather than treating strong emotion merely as individual-level noise, we position it as a potential diagnostic signal of structural friction. This perspective guides our inquiry around three interrelated questions: (1) How do citizens' emotional intensity profiles vary across major governance themes in a digital complaint system? (2) In which domains do we observe disproportionate concentrations of extreme emotional expressions, and what linguistic markers of perceived procedural injustice characterize these clusters? (3) How do cross-domain linguistic patterns reflect the cognitive gap between citizens' integrated problem representations and segmented institutional categories, and what might this imply for perceived procedural clarity?

By coupling large-scale topic modeling with emotion analysis, this study examines how collective emotional patterns may function as diagnostic signals of coordination bottlenecks. More broadly, the study contributes to the United Nations' Sustainable Development Goal 16 (Peace, Justice and Strong Institutions) by highlighting how institutional design may matter for perceived procedural fairness and legitimacy in digitally mediated citizen-state interactions.

## 2 Materials and methods

We applied a five-stage analytical workflow. First, we conducted dimensional emotion annotation (polarity, intensity, and submission type) as a proxy for citizens' psychological appraisals. Second, BERTopic modeling identified thematic structures across 76,447 submissions. Third, cross-topic keyword extraction and network visualization detected domain overlaps reflecting fragmented institutional responsibilities. Fourth, emotion–topic coupling measured systematic variation in emotional intensity across governance domains. Finally, TF-IDF weighting of extreme-intensity submissions identified linguistic risk markers associated with perceived procedural injustice. This workflow links large-scale text mining with psychological theory on appraisal and control. The study did not require ethical approval. According to China's Ethical Review Measures for Life Sciences and Medical Research Involving Human Beings (effective 2023), this study is exempt from ethical review because it (i) utilizes lawfully obtained public data generated through observation without interfering with public behavior, and (ii) uses anonymized information data. No individually identifiable data were collected, processed, or reported beyond what is publicly accessible on the platform.

### 2.1 Data collection and pre-processing

This study analyzed citizen submissions from “Min Hu Wo Wei” (People Call, We Respond), a government-operated institutional feedback platform in Zhejiang Province, China. The platform integrates multiple official channels, including municipal hotlines, online inquiry systems, and community observation portals. Prior research using BERTopic on this platform has demonstrated the method's ability to extract structured demand categories from citizen submissions (Tang et al., 2024). All submissions must receive responses from designated administrative units within a regulated time window, creating a structured two-way communication mechanism that differs from public social media.

Using web scraping tools, we collected all publicly available submissions from the “Min Hu Wo Wei” platform between July 2023 and June 2024. Each record contains the submission title, main content, submission date, responding administrative unit, response content, and response date. After removing duplicates and incomplete entries, a total of 76,447 valid submissions remained.

Preprocessing involved three steps. First, regular expressions were applied to remove non-Chinese characters, special symbols, and standalone date strings. Second, named entity recognition was used to identify domain-specific terms (e.g., terms used for housing assistance or community medical facilities), which were added to a custom dictionary and used for word segmentation with Jieba to improve accuracy. Third, stopwords were removed by merging multiple stopword lists and excluding courtesy expressions frequently found in citizen letters (e.g., common greetings addressed to officials) that carry little semantic value for content analysis. All data are publicly accessible and anonymized; no personally identifiable information beyond what is already available on the platform was included in the analysis.

### 2.2 Dimensional emotion annotation

Emotional expression in citizen submissions was treated as a proxy for underlying psychological appraisals in digitally mediated state–public interactions. Following appraisal theory, emotional strength reflects cognitive evaluations of relevance, fairness, and controllability rather than objective issue severity alone (Lazarus, 1991). At the textual level, these appraisals manifest as dimensional emotional patterns, which can be observed through polarity, intensity, and communicative orientation.

Sentiment annotation followed a three-dimensional model: polarity (positive, neutral, negative), intensity (mild, strong, extreme), and submission type (inquiry, suggestion, complaint, other). In our socio-psychological framework, polarity captures the valence of the citizen's appraisal, while intensity serves as a linguistic proxy for psychological arousal and diminished perceived control. The submission type reflects the behavioral orientation expressed in the narrative (e.g., passive request vs. active escalation). By operationalizing emotion through these dimensions, we transform unstructured text into observable signals of citizens' appraisal patterns in digitally mediated complaint contexts.

A direct measurement approach—administering validated appraisal scales (e.g., perceived control, perceived fairness) to citizens at the time of submission—would provide stronger construct validity. However, the archival and large-scale nature of the data precludes contemporaneous self-report measurement. Text-based annotation thus represents a methodological trade-off: it sacrifices precision in measuring individual psychological states in exchange for ecological validity, population-level coverage, and the ability to detect systematic patterns across 76,447 submissions that would be inaccessible through survey designs. We treat the resulting measures as proxies for aggregate appraisal tendencies rather than direct assessments of individual psychological states.

Automated annotation was conducted using the Qwen-Turbo-2024-11-01 large language model, selected for its performance on Chinese administrative complaint texts and its ability to recognize procedural-emotion markers (e.g., “rectification,” “handling,” “delay”). A structured prompt returned standardized JSON outputs. To assess reliability, we drew a stratified sample of 300 submissions covering variation in all dimensions and employed two independent human coders. Inter-coder reliability was substantial to high (mean Cohen's κ = 0.79). Human–machine agreement reached 93.6% for submission type (κ = 0.89), 86.1% for polarity (κ = 0.72), and 85.3% for intensity (κ = 0.76). Automated annotation was therefore applied to the full corpus.

These three dimensions provide operational measures of citizens' psychological responses embedded in complaint texts, enabling a psychologically informed interpretation of emotional patterns at scale while avoiding individual-level inference.

### 2.3 Topic modeling with BERTopic

We applied BERTopic (Grootendorst, 2022) to identify thematic structures within citizen submissions. The workflow consisted of four steps. First, we generated 384-dimensional sentence embeddings using the “paraphrase-multilingual-MiniLM-L12-v2” transformer model implemented in the Sentence-BERT architecture (Reimers and Gurevych, 2019). Second, dimensionality reduction was performed using UMAP (McInnes et al., 2018) to preserve local semantic relationships in the embedding space while enabling efficient clustering. Third, we applied HDBSCAN (McInnes et al., 2017) to identify dense regions and group similar documents into candidate topics. Parameters for both UMAP and HDBSCAN were iteratively tuned to improve topic coherence and avoid excessive fragmentation. Fourth, we used class-based TF-IDF (c-TF-IDF) to obtain interpretable topic representations by treating all documents in a cluster as an aggregated document and extracting terms distinctive to each cluster.

Initial modeling with default parameters and outlier reduction produced 70 topics. Visual inspection of the 2D topic distribution (Supplementary Figure S1) revealed substantial clustering and semantic overlap across multiple regions, indicating opportunities for consolidation. To improve interpretability and reduce redundancy, we iteratively adjusted the min_topic_size parameter, ultimately obtaining 38 second-level topics with improved coherence and reduced fragmentation (Supplementary Figure S2). Further consolidation into 10 first-level domains was guided by a joint review of hierarchical clustering visualizations (Supplementary Figure S3), topic similarity matrices (Supplementary Figure S4), topic keywords, and representative submissions. The dendrogram and similarity matrix helped identify semantically proximate topics and potential boundary cases, while the keyword lists and representative submissions were used to interpret the substantive focus of each topic. Two researchers reviewed these materials and then collaboratively assigned domain labels through structured discussion. When a topic's keywords included cross-domain process or remedy terms such as “application,” “processing,” and “refund,” assignment followed citizens' substantive demands and the administrative system most directly responsible for handling them. For example, the two provident fund topics cluster with “qualification and certificate processing” and “residence permits and backyard poultry” in Supplementary Figure S3, and all four share “application” and “processing” among their top 30 keywords; they were nevertheless assigned to different domains based on representative submissions and administrative responsibility. Similarly, “training institutions and refunds” appears in the same dendrogram branch as “event ticket refunds” and “e-commerce platform disputes,” and all three include “refund” among their top 30 keywords; it was assigned to education and training because the disputes concern training services and fees. Shared process- or remedy-related vocabulary was not treated as sufficient for consolidation.

### 2.4 Cross-topic keyword extraction and network visualization

To identify governance domains where citizen concerns overlap, we analyzed keyword co-occurrence patterns across topics. The top 30 keywords with the highest c-TF-IDF weights were extracted from each of the 38 second-level topics, yielding 778 unique keywords after deduplication. Keywords appearing in at least two second-level topics spanning different first-level domains were classified as cross-topic bridging terms, resulting in 156 such keywords (20.1% of all representative topic keywords).

To visualize connectivity patterns, an interactive network was constructed using PyVis in Python, with first-level topics positioned at the periphery and keywords at the center. Edges connect keywords to topics when the keyword appeared among the representative terms for any second-level topic within that domain. The force-directed layout positions highly connected keywords centrally, revealing which terms bridge multiple governance areas. Psychologically, this network provides a text-based approximation of how citizens articulate lived problems across institutional boundaries, in contrast to the segmented categories used in administrative processing.

### 2.5 Emotion–topic coupling analysis

To assess how emotional expression varies across governance domains, an emotion–topic matrix was constructed that relates sentiment polarity and intensity to the ten first-level topics identified through topic modeling. This design enables a comparative view of where emotional escalation is most concentrated and how different forms of citizen expression—complaints, inquiries, or suggestions—cluster within specific thematic areas. The resulting distributions were visualized through bar charts and a heatmap to identify domains characterized by disproportionately high rates of negative or strong-intensity emotion.

### 2.6 Risk keyword identification

To identify the linguistic features associated with extreme emotional expression, we isolated the subset of submissions classified as exhibiting the highest sentiment intensity. Term weighting was then performed using the TF-IDF algorithm, which highlights words that are disproportionately salient within this subset relative to the full corpus, rather than merely frequent. This weighting scheme is widely used in text mining for detecting distinctive lexical markers in focal text segments (Salton and Buckley, 1988; Ramos, 2003). The ranked output was subsequently grouped into semantic categories to identify recurring patterns among the high-weight terms. Each term was interpreted in the context of the submissions in which it appeared, with full texts consulted where usage was ambiguous. Two researchers reviewed the terms, discussed groupings, and consolidated them into broader categories.

## 3 Results

### 3.1 Descriptive overview of citizen submissions

The dataset contained 76,447 valid citizen submissions from July 2023 to June 2024, with a daily average of approximately 210 submissions. Keyword frequency analysis of the main text fields showed that high-frequency terms were highly concentrated around daily living conditions and administrative handling. Table 1 presents the frequency distribution of the top keywords extracted from citizen submissions.

**Table 1:** Frequency of top keywords in citizen submissions (*N* = 76, 447).

Keyword	Frequency	Keyword	Frequency	Keyword	Frequency
Handling	11,435	Concerned departments	5,414	Property management	3,832
Impact	11,247	Government	5,263	Safety hazard	3,671
Residential community	8,939	Related departments	5,207	Processing	3,595
Homeowner	8,546	Hangzhou	5,053	Application	3,575
Solve	8,440	Delivery	4,392	Phone call	3,566
Situation	7,582	Developer	4,349	Related	3,467
Cause	6,865	Work	4,287	Personnel	3,373
Discover	5,861	Notification	4,210	Resident	3,325
Time	5,858	Company	4,112	Contact	3,259
Complaint	5,580	Suggestion	3,977	Subdistrict office	3,227

These keywords show that citizen submissions were primarily concerned with residential environments, community governance, service delivery, and administrative processing. Frequent mentions of “residential community”, “homeowner”, “developer”, “property management”, and “safety hazard” indicate the prominence of housing-related issues. References to “relevant departments”, “government”, and “related departments” reflect repeated attention to administrative responsiveness and departmental coordination. Terms such as “impact”, “cause”, and “situation” commonly appeared in descriptions of problem contexts and consequences in daily life.

### 3.2 Sentiment distribution patterns

**Figure 1:**
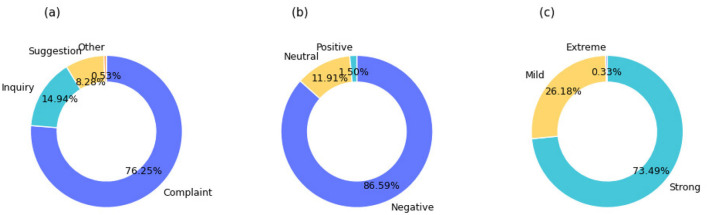
Distribution of **(a)** submission types, **(b)** sentiment polarity, and **(c)** sentiment intensity.

Figure 1 shows the distribution of (a) submission types, (b) sentiment polarity, and (c) sentiment intensity across the 76,447 citizen submissions.

With respect to submission type, complaints accounted for 58,291 submissions (76.25%), followed by inquiries (11,421; 14.94%) and suggestions (6,329; 8.28%). Other types constituted a small share (406; 0.53%). The distribution indicates that the platform was mainly used to register complaints and problem reports, whereas consultative and advisory uses were relatively less frequent.

Sentiment polarity showed a highly skewed distribution toward negative evaluations. Of the 76,447 submissions, 66,199 (86.59%) were classified as negative, 9,102 (11.91%) as neutral, and 1,146 (1.50%) as positive. The polarity pattern indicates that citizen submissions were dominated by problem-oriented responses rather than neutral or positive feedback.

Sentiment intensity was further categorized into mild, strong, and extreme levels. Strong-intensity submissions represented the largest share (56,182; 73.49%), followed by mild-intensity submissions (20,016; 26.18%). A distinct subset of 249 submissions (0.33%) was classified as extreme. Although small in proportion, the extreme-intensity subset reflects cases with highly charged language or crisis-related expressions.

### 3.3 Topic structure and cross-topic keywords

BERTopic modeling yielded 10 first-level domains and 38 second-level topics. Table 2 summarizes the distribution of submissions across first-level topics and lists example second-level themes.

**Table 2:** Distribution of submissions across first-level topics (*N* = 76, 447).

First-level topic	n	%	Example second-level topics
A. Housing and property management	19,746	25.83	Real estate development and property management; facility safety; community construction and daily life; residence permits and backyard poultry
B. Transportation and mobility	14,794	19.35	Traffic signals and road safety; parking and violations; metro planning and construction; public transport services; ride-hailing and airport access; metro passages and hotel services
C. Environmental health and sanitation	12,901	16.88	Waste management; noise pollution; public facilities; street vendors and city image; small advertisements; biodiversity and ecological protection
D. Consumer rights and e-commerce	6,913	9.04	E-commerce platform disputes; minors' game spending and refunds; event ticket refunds; beauty and prepayment cards
E. Labor rights and social security	6,833	8.94	Wage payment; social insurance and retirement; housing loans and provident fund policies; provident fund services and information queries
F. Education and training	5,535	7.24	School zoning and enrollment; qualification and certificate processing; training institutions and refunds
G. Legal services and government affairs	4,389	5.74	Legal cases and litigation; tax issues and social insurance contributions; enterprise services and government communication
H. Health and medical services	2,679	3.50	Medical services and reimbursement; fitness safety and facility management; hotel services and food safety
I. Public safety and emergency management	1,784	2.33	Fire safety and passage management; historical commemoration and air-raid sirens; neighborhood disputes and mediation
J. Public utilities and services	873	1.14	Telecommunications services; gas supply and installation

The five largest domains—housing and property management (25.83%), transportation and mobility (19.35%), environmental health and sanitation (16.88%), consumer rights and e-commerce (9.04%), and labor rights and social security (8.94%)—together covered 61,187 submissions (80.04%). Within housing and property management, “Real Estate Development and Property Management” alone contained 14,641 submissions (19.15% of all cases). In transportation and mobility, “Traffic Signals and Road Safety” and “Parking Management and Traffic Violations” accounted for 7,469 (9.77%) and 3,909 (5.11%) submissions, respectively. In environmental health and sanitation, “Waste Management and Garbage Disposal” comprised 5,657 cases (7.40%) and “Noise Pollution and Residential Life” 3,829 (5.01%).

**Figure 2:**
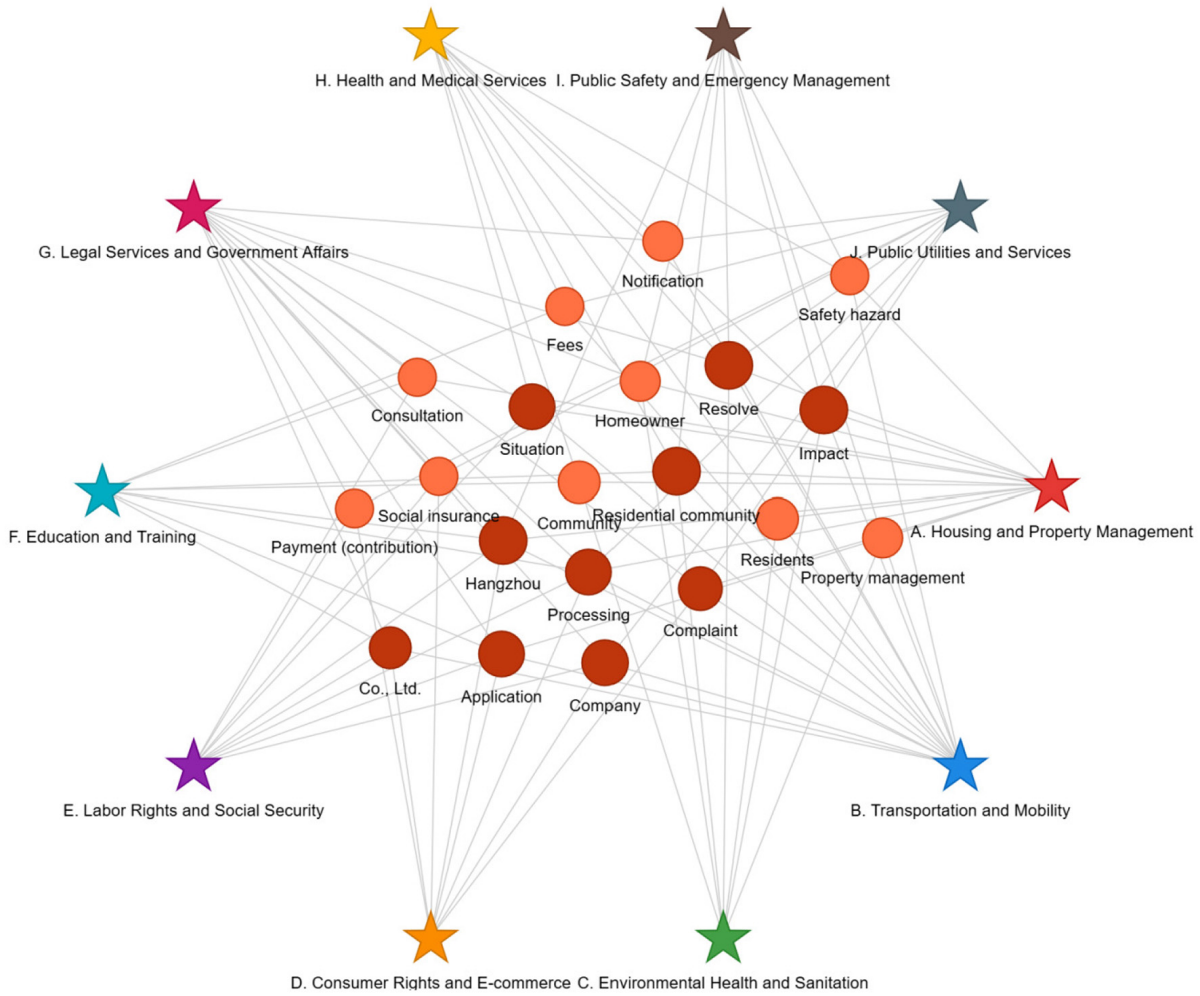
Network of topic-keyword connections.

To visualize how citizen-referenced concepts connect across administrative domains, a keyword–topic network was constructed (Figure 2). The ten first-level topics are positioned at the periphery, while the most recurrent keywords occupy the center. The network shows a dense and multi-directional structure, indicating that citizen submissions often articulate problems through shared experiential terms that cut across institutional boundaries.

A small set of keywords anchors the structure. Terms such as “residential community,” “processing,” “complaint,” “homeowner,” and “safety hazard” connect simultaneously to multiple governance domains. “Residential community” sits at the center of the network and links to nearly all major service areas, including housing management, mobility safety, environmental sanitation, school admission, healthcare facilities, and fire protection. This pattern is consistent with the spatial logic of daily life, in which multiple public functions converge within the same physical context of the neighborhood and may be narrated as a single situation.

Administrative process terms occupy the same position. Words such as “processing” and “application” link to topics including social insurance enrollment, education certificates, housing permits, and consumer complaints. Their high connectivity highlights a recurring point of procedural coordination across distinct policy areas. Other highly connected terms—“homeowner,” “property management,” “resident”—appear at the intersection of housing disputes, environmental management, legal recourse, and safety enforcement. The same actors face different regulatory regimes simultaneously. The network therefore does not present separate issue clusters. It depicts an integrated semantic space organized around a small number of bridging concepts. These concepts are not tied to a particular functional department; rather, they correspond to the ways citizens articulate experiences that intersect with multiple service delivery lines simultaneously. The resulting topological structure indicates that citizen-referenced vocabulary is organized around shared spatial and social contexts rather than single functional domains.

### 3.4 Emotion–topic coupling patterns

**Figure 3:**
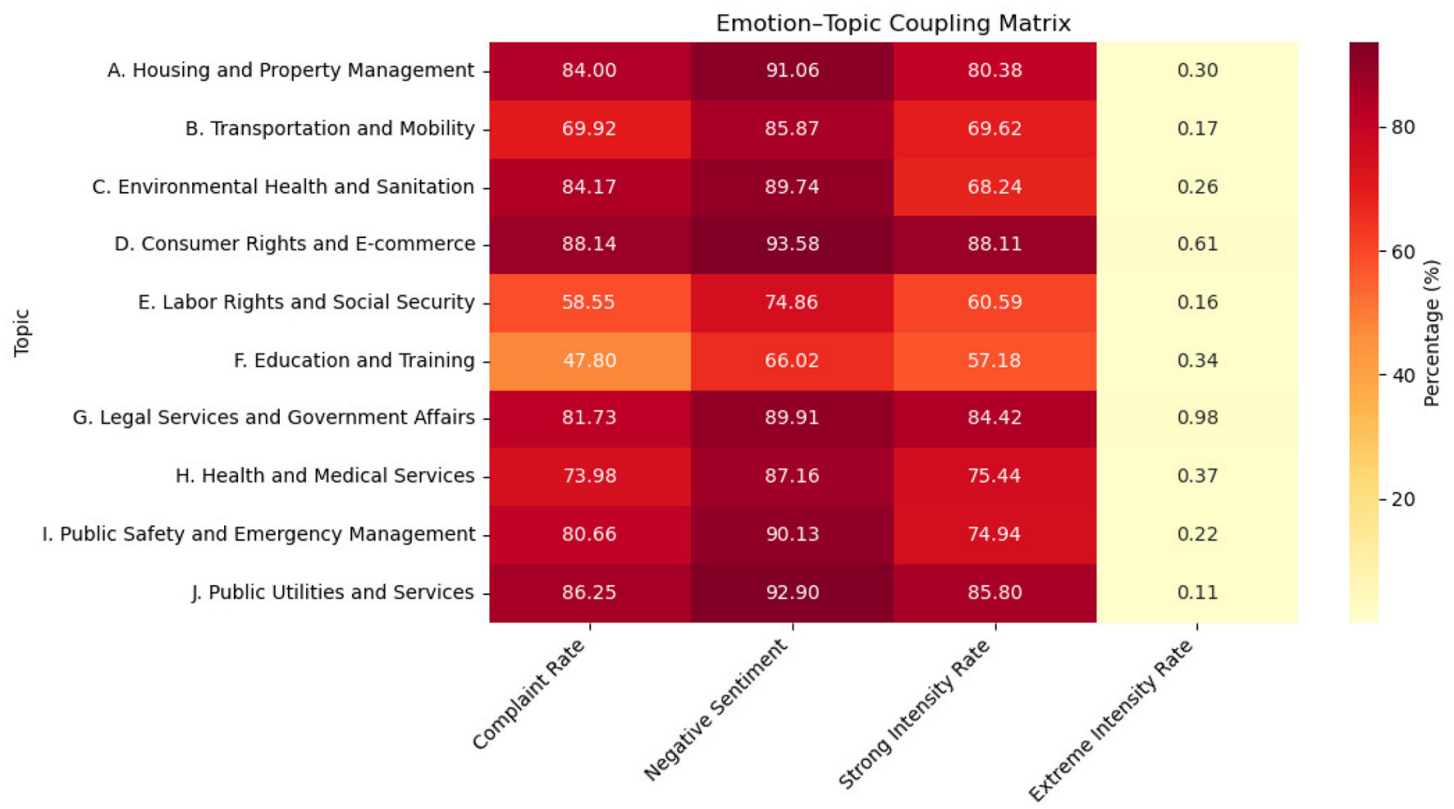
Emotion-topic coupling matrix.

An emotion–topic matrix was constructed to examine how polarity, intensity, and complaint patterns vary across the ten governance domains (Figure 3). Negative sentiment is prevalent in all areas. Consumer rights and e-commerce, public utilities and services, housing and property management, and public safety show rates above 90%. These domains involve issues directly affecting daily living conditions and typically reference specific service providers or organizations in submission texts. By contrast, education and training and labor rights exhibit lower complaint rates and milder sentiment intensity. These domains are typically governed by standardized administrative procedures with defined timelines and eligibility criteria.

Strong-intensity expressions dominate several domains. Consumer rights and e-commerce, public utilities and services, and legal services and government affairs show particularly high proportions of reinforced emotional language. Notably, high emotional intensity in these domains does not correspond straightforwardly to long-term welfare relevance: labor rights and education, whose substantive content concerns long-horizon individual welfare, show the mildest intensity profiles. Housing and property management shows a similar pattern. The concentration of family assets and long time horizons make each decision consequential.

Table 3 presents six translated excerpts that illustrate how these intensity levels correspond to different expressive forms across governance domains.

**Table 3:** Citizen submissions by emotional intensity and governance domain.

Emotion intensity	Domain	Excerpt (translated)	Expressive features
Mild	Labor rights and social security	“Hello, may I ask how it should be handled if a company has not paid the housing provident fund? If the company makes supplementary contributions, how are the amount and ratio calculated?”	Courteous inquiry; procedural question; neutral tone
Mild	Education and training	“During the application process, I cannot change the bank and account information myself, and the default account is no longer in use.”	Brief factual report; no intensifiers; concrete administrative obstacle
Strong	Transportation and mobility	“The new school term is about to start. Our compound has nearly five thousand residents, and several hundred pupils must travel to the primary school, yet both available routes are hazardous. Please build a simple footpath so the children can go to school safely and come home safely.”	Urgency tied to children's safety; collective scale; constructive request
Strong	Consumer rights and e-commerce	“Contradictory conclusions dozens of times! Simply absurd! A trivial matter has dragged on for more than 2 years and still has not been resolved. Whoever is responsible for this case should write down their name.”	Exclamation; sarcasm; prolonged frustration; demand for accountability
Extreme	Housing and property management	“The facade is rough and uneven to an extreme degree. This is a construction defect. We strongly ask the government department to intervene, require the existing coating to be removed and redone, or upgrade it to integrated panels, and give homeowners a satisfactory response.”	Intensified evaluation; direct demand for government intervention; collective homeowner framing
Extreme	Environmental health and sanitation	“Every morning from six o'clock, the street-cleaning truck plays loud music while working for three to 4 h. It is very, very disturbing, whether on weekdays or weekends, and seriously affects residents' daily life. Even from the 25th floor it can be heard clearly. Could the music be turned off during morning cleaning?”	Repetition of intensity; daily-life disruption; concrete remedial request

Extreme-intensity cases are rare across all domains. Peaks appear mainly in legal services and government affairs, consumer rights and e-commerce, and education and training. Qualitative inspection of these extreme cases reveals that many describe multiple prior attempts to resolve the issue through administrative channels, referrals across departments, and a shift in language from specific requests to broader appeals. In summary, emotional intensity varies systematically across governance domains. Higher intensity concentrates in domains characterized by multi-actor accountability arrangements, while domains with standardized, single-authority procedures show milder profiles. This distributional pattern is examined further in the Discussion.

### 3.5 Extreme emotion risk patterns

**Figure 4:**
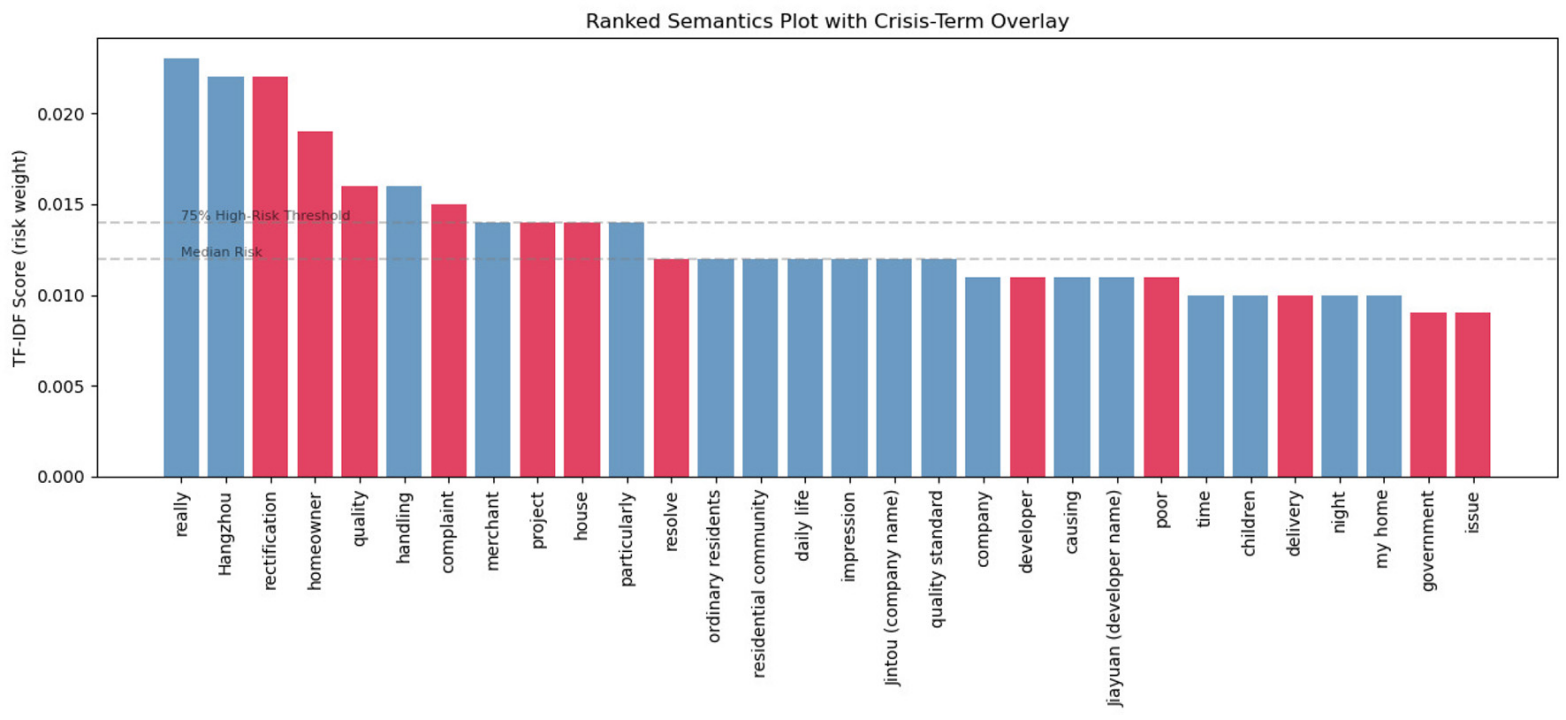
Ranked TF-IDF weights of extreme-emotion keywords with crisis-term overlay.

To examine the semantic structure of extreme emotional expressions, TF-IDF weights were calculated for the 249 submissions classified as exhibiting the highest intensity. The top-ranked terms are distinctive within this subset rather than merely frequent across the full corpus (Figure 4). TF-IDF therefore highlights the lexical markers that carry disproportionate weight in extreme cases. Table 4 defines these semantic categories and provides example items from the TF-IDF ranking.

**Table 4:** Semantic categories for high-weight terms in extreme-intensity submissions.

Category	Definition	Example terms
Emphatic intensifiers	Degree adverbs that amplify the force of statements	“really,” “particularly”
Procedural action terms	Vocabulary of administrative processing and case handling	“rectification,” “handling,” “complaint,” “resolve”
Objects of dispute	Concrete referents of the underlying grievance	“project,” “house,” “quality”
Collective identity markers	Terms situating the sender within a wider affected group	“homeowner,” “ordinary residents”
Everyday-life context terms	References to daily routines disrupted by the problem	“daily life,” “children,” “night”
Administrative responsibility references	Mentions of government or responsible units	“government,” “multiple departments”
Crisis expressions	Direct references to personal crisis or self-harm	“suicide,” “jumping”

Figure 4 displays the highest-weighted terms. The pattern does not revolve around isolated crisis language. Instead, the high-weight vocabulary combines several distinct components: emphatic adverbs (“really,” “particularly”), procedural language (“rectification,” “handling,” “complaint”), concrete objects of dispute (“project,” “house,” “quality”), and collective identity markers (“homeowner,” “ordinary residents”). Procedural terms outnumber event-specific or crisis-related terms among the highest-weighted keywords. Terms such as “rectification,” “handling,” “complaint,” and “solution” rank among the highest-weighted items, indicating that the distinctive vocabulary of extreme-intensity submissions centers on administrative processing rather than the substantive content of disputes. Property-related terms carry clear personal stakes and correspond to domains where responsibility is highly dispersed. Contextual terms such as “daily life,” “children,” and “night” co-occur with procedural vocabulary, indicating that extreme-intensity language appears alongside descriptions of ongoing routine disruptions rather than discrete crisis events. The convergence of procedural action terms, property-related vocabulary, and dispersed accountability references forms a distinctive semantic cluster in extreme-intensity submissions. The theoretical implications of this pattern are examined in the Discussion.

Three linguistic elements converge in the extreme-intensity subset: stalled procedural terms, property-related insecurity vocabulary, and references to dispersed accountability across departments. This convergence parallels the emotion–topic coupling results, where housing disputes, consumer rights, and legal affairs showed the highest proportions of extreme emotion. Crisis-related vocabulary (e.g., references to self-harm) appears only in the thin tail of the TF-IDF distribution, whereas administrative processing language constitutes the core semantic structure.

## 4 Discussion

### 4.1 Procedural entrapment and the stake-intensity asymmetry

The divergence between topic domains and emotional escalation suggests that emotional intensity may depend less on the objective magnitude of issues than on how citizens appraise their administrative encounters. Appraisal theory posits that emotional strength reflects evaluations of relevance, injustice, and controllability rather than material severity alone (Lazarus, 1991). Classic organizational research, including Affective Events Theory (Weiss and Cropanzano, 1996), similarly shows that routine or low-stakes events can evoke intense emotional reactions when they are interpreted as unfair, opaque, or obstructive (Weiss et al., 1999; Barclay et al., 2005). Thus, citizens react not only to what is at stake, but to whether they can meaningfully influence the pathways through which their issues are processed—a core tenet of perceived procedural justice (Tyler, 2006).

The empirical patterns reported above present a notable asymmetry. Domains that concern citizens' long-term welfare, such as labor rights and education, show the mildest emotional profiles. Emotional intensity concentrates instead in domains such as housing disputes and consumer rights, where multiple actors may respond but no single authority holds integrative responsibility. The stakes in these domains are themselves substantial: housing concentrates the largest financial asset most households hold, and consumer disputes involve immediate realized losses. Extreme-intensity submissions are linguistically dominated by procedural action terms describing administrative processing, with crisis vocabulary confined to the thin tail of the distribution. These observations motivate the theoretical interpretation that follows.

A plausible explanation for the emotional escalation observed in our data lies in procedural structure rather than the magnitude of material consequences alone. Standardized administrative domains—such as pension claims or school admissions—operate through clear timelines, identifiable authority, and predictable decision points. Even when delays occur, citizens can discern who is responsible, supporting a secondary appraisal of controllability (Lazarus, 1991). In contrast, domains like housing disputes and consumer rights are typically embedded in fragmented accountability arrangements. Multiple actors—developers, subdistrict offices, platform operators, and regulatory departments—may respond, yet no single authority holds final, integrative responsibility. Such dispersed accountability undermines the conditions for effective accountability assessment (Bovens, 2007) and creates procedural ambiguity. Other factors may also contribute to this gradient. The keyword “labor arbitration” appears among the top 30 c-TF-IDF terms in the wage-payment topic, suggesting that some disputes in this domain may be pursued through formal arbitration or litigation channels. Private counterparties, including developers, employers, merchants, and platform operators, add actors to the chain of responsibility and complicate attribution in housing, labor, and consumer disputes. Citizens may also react more strongly to discrete, realized losses such as a defective dwelling or an unrefunded payment than to long-horizon welfare consequences, a pattern consistent with prospect theory's emphasis on the psychological salience of losses (Kahneman and Tversky, 1979).

This ambiguity may make both problem-focused and emotion-focused coping more difficult to sustain. According to the transactional model of stress and coping (Lazarus and Folkman, 1984), individuals typically employ problem-focused coping (addressing the source) when situations appear changeable, or emotion-focused coping (managing distress) when circumstances seem immutable. In highly fragmented complaint systems, citizens cannot easily employ problem-focused coping because definitive responsibility is evasive, yet they cannot achieve closure through emotion-focused coping because institutional responses constantly signal that action should be possible. This dual coping impasse is theoretically consistent with sustained emotional escalation, as neither strategy offers a pathway to psychological closure (Lazarus and Folkman, 1984). Experimental research further confirms that ambiguity in procedures amplifies anger and negative affective reactions because individuals cannot determine whether authorities are acting fairly or with intentional disregard (De Cremer et al., 2008).

The procedural entrapment dynamic introduced above manifests distinctly in these data. While it shares conceptual roots with learned helplessness—where individuals develop expectations of uncontrollability and passively withdraw (Seligman, 1972)—procedural entrapment manifests quite differently. Instead of passive resignation, individuals in these contexts may experience a contradictory state of active exhaustion. Institutional pathways remain formally open, requiring continuous self-advocacy and engagement. Yet functionally, these pathways may operate through circular referrals and fragmented processing that are associated with depletion of psychological resources without advancing toward resolution.

Table 5 illustrates this dynamic in three cases from the corpus. Each began with a specific grievance, passed through multiple units that disclaimed responsibility or gave inconsistent responses, and ended with the citizen appealing to law, official duty, or government responsiveness.

**Table 5:** Three cases illustrating prolonged procedural engagement.

	Case 1	Case 2	Case 3
Domain	Housing and property management	Consumer rights and e-commerce	Environmental health and sanitation
Initial grievance	Neighbor enclosing shared corridor, blocking ventilation and light	Suspected counterfeit goods from unauthorized online seller	Reported nighttime construction noise exceeding legal limits
Parties contacted	Property company, municipal hotline, fire department, administrative enforcement, subdistrict office, village committee	E-commerce platform, brand distributor, district market regulation bureau, merchant-locality bureau	Subdistrict enforcement squad, municipal environmental authority
Handoff pattern	Each unit confirmed the problem or its illegality but disclaimed enforcement power and redirected the case	Platform provided no substantive handling; bureau responded with a text message after 5 weeks; platform “repeatedly provided wrong addresses” for cross-jurisdiction filing	Squad cited a construction permit; oral and written replies contradicted each other; later petitions were routed back to the same squad
Citizen framing	“Shifted the matter to one another, gave no response, and dragged out the time”; cites Civil Code articles; demands departments “genuinely handle matters for the people”	Describes repeated attempts through four parties with no resolution route available	“Has dragged on for months without resolution”; questions the motives of offices involved
Status at time of writing	Enclosure completed during referral process	Not resolved	Not resolved

In each case the citizen first approached the unit that appeared responsible; when that unit disclaimed authority or gave no substantive response, the search widened to hotlines, bureaus, and higher authorities. Each new contact produced activity but not resolution: the fire department confirmed the violation yet reported no enforcement power, the market regulation bureau answered after 5 weeks with a text message, and the enforcement squad's telephone reply did not match its written one. The closing language reaches past the original request: Case 1 quotes the Civil Code and demands that departments “genuinely handle matters for the people,” and Case 3 questions the motives of the offices involved. The grievance has expanded from the original issue to the handling process itself.

Linguistic evidence from the extreme-intensity subset is consistent with this interpretation. The most distinctive terms in these submissions are procedural action verbs (“rectification,” “handling,” “transfer”) paired with references to “multiple departments”; crisis expressions (“suicide,” “jumping”) appear rarely and are concentrated in the thin tail of the distribution. This vocabulary profile is consistent with a reading in which high-intensity expression centers on procedural indeterminacy: situations in which institutional activity is visible yet final accountability cannot be located. Complaint submissions, however, are mediated and sometimes strategic texts. Research on mediated communication suggests that message form can reflect platform conditions and communication norms as well as sender intent (Hancock et al., 2020), and work on government-citizen communication in China distinguishes informational-cognitive aspects of message quality, such as clarity and ease of response, from expressive-constitutive aspects, such as respectfulness, trust, urgency, and empathy (Zhang and Nie, 2026). The procedural vocabulary can therefore be read in overlapping ways: as exhaustion after repeated unsuccessful engagement, as strategic intensification aimed at securing administrative attention, or as adaptation to the action-oriented vocabulary of administrative processing. The corpus also displays recognizable rhetorical registers: emphatic and exclamatory language, deferential openings and gratitude closings, citations of statutes and national standards that anchor claims in official commitments (O'Brien, 1996), and collective framing in the voice of homeowners, villagers, or ordinary residents, a form that prior experimental work has linked to greater official responsiveness when it signals potential collective action (Chen et al., 2016). High-intensity language appears especially in submissions that narrate prolonged engagement, stalled handling, and dispersed responsibility, and it is particularly concentrated in governance domains where accountability is fragmented. The evidence is therefore diagnostic in scope: it identifies pathways that citizens describe as difficult to navigate, while disentangling the affective from the strategic sources of intensity would require designs that observe repeated interactions over time.

### 4.2 Cross-domain patterns and institutional fragmentation

The network of cross-domain keywords highlights a recognizable gap between how citizens experience governance problems and how public organizations are structured to process them. In the semantic network, bridging terms—such as “residential community,” “processing,” and “resident”—link multiple thematic domains rather than remaining confined to a single policy area. These terms typically appear within localized narratives of daily life, where housing quality, mobility safety, sanitation, and public safety coexist in a single neighborhood context. Psychologically, this structural pattern suggests that citizens articulate issues through primary frameworks (Goffman, 1974) organized around integrated spatial and social experiences of their neighborhood, rather than through the functional categories used in administrative processing. One urban submission frames what it calls “several livelihood problems” in a single residential compound: an unpaved road section that produces traffic noise, amplified square dancing in the adjacent park, curbside parking that blocks entry to the compound and would obstruct fire engines, and the fees and conduct of the property management company, addressing the subdistrict office, the community, and the traffic police together. A village submission describes one road section where village-road access and traffic safety, illegal parking, weeds, garbage, insect infestation, and a fetid drainage ditch accumulate after coordination between the village committee, the highway authority, and the subdistrict office failed. In both texts one lived situation spans administrative functions that process its components separately; the village writer names the result a zone that “none of the three manages.” In the Chinese context, this holistic articulation may also be consistent with broader culturally shaped expectations that government-facing problems should be addressed in an integrated manner (Shi, 2015). What the data show directly are integrated problem narratives on the citizen side and segmented processing categories on the institutional side. This gap is also linked to concrete platform and institutional arrangements: the platform merges municipal hotlines, online inquiry systems, and community observation portals into a common intake, while responses are assigned to designated functional units within regulated time windows. Such a design allows citizens to enter problems as integrated narratives but processes them through functional categories, and the evidence in this study bears on this institutional configuration.

This pattern reflects what we term a spatial–functional misalignment. The bridging terms themselves are partly administrative-process vocabulary shared across the platform, so the reading rests jointly on the network structure, the neighborhood terms at its center, and the integrated narratives discussed above. Citizens approach platforms with unified representations of localized problems, yet institutions process these demands through segmented administrative silos. Prior public administration research terms this structural pattern bureaucratic fragmentation, where agencies address cross-cutting issues sequentially or through complex referral chains (Lekkas and Souitaris, 2023). From a socio-psychological perspective, however, this misalignment may create additional cognitive and psychological demands for citizens. Translating a holistic, lived experience into fragmented bureaucratic categories requires individuals to navigate opaque jurisdictional boundaries.

When handoffs stall and responsibility diffuses across these boundaries, this structural friction is often appraised by citizens as a lack of procedural justice. As previous research on cross-agency collaboration notes, coordinated action across fragmented environments is the exception rather than the default, often entailing high transaction costs and ambiguous responsibility structures (Scott and Merton, 2021), and requiring explicit governance capacities that go beyond information sharing alone (Gasco-Hernandez et al., 2022). Thus, the cross-domain bridging vocabulary observed in our data does not merely describe complex social problems; it offers a text-based approximation of the psychological cost of institutional design. It visually highlights intersections where coordination deficits may be linked to reduced procedural clarity and a diminished sense of agency.

### 4.3 Toward emotional responsiveness

Research on digital governance typically evaluates responsiveness in procedural terms, prioritizing metrics such as reply speed, case closure rates, and administrative throughput (Nie and Wang, 2023; Tavares et al., 2022). However, our emotion–topic patterns indicate that responsiveness also has an important psychological dimension. Extreme emotional expressions, while statistically rare, concentrate disproportionately in domains characterized by dispersed authority. The vocabulary of these extreme cases emphasizes “handling,” “delay,” and “multiple departments” over sudden crisis terms; these submissions often narrate prolonged engagement with unresolved procedural pathways. Digital platforms, characterized by lower expression costs and reduced self-censorship (Suler, 2004), effectively surface these cumulative frustrations earlier than traditional offline channels.

In this context, the findings point toward what might be termed emotional responsiveness—a governance capability focused on addressing the structural sources of emotional intensity rather than merely managing its visible expressions. Instead of treating anger as individual-level noise or a public relations issue to be moderated, institutions can use aggregated emotional patterns as diagnostic signals. Research on consumer complaint behavior has shown that emotional expectations and perceived fairness of complaint-handling processes shape citizens' subsequent evaluations and behavioral responses (Wang et al., 2022; Tax et al., 1998). These collective emotional signals point to domains in which responsibility diffusion and obstructed resolution pathways may be associated with reduced perceived control. In practical terms, they highlight institutional intersections where limited coordination capacity may contribute to reduced procedural clarity and, in turn, diminished perceived control. From a psychological standpoint, addressing these specific systemic bottlenecks may enhance perceived procedural fairness and predictability, which are central mechanisms by which individuals evaluate institutional legitimacy (Tyler, 2006). These dynamics align with recent findings on information integration in government, where institutional hierarchies and procedural fragmentation impede coordinated approaches to cross-domain problems (Zogheib and Mahetaji, 2024).

Ultimately, this psychological perspective aligns deeply with the United Nations' Sustainable Development Goal 16, which emphasizes the development of effective, accountable, and transparent institutions. By treating collective emotional data as one source of information for institutional design, public administrations can move beyond superficial procedural compliance. Identifying and addressing the structural conditions associated with escalation may help reduce collective emotional intensity while supporting stronger perceptions of justice and institutional trust in digitally mediated state–public interactions.

### 4.4 Limitations and future research

While this study offers a novel computational perspective on citizen-state interactions, several limitations point to fruitful avenues for future research. First, we rely on linguistic expressions as text-based proxies for cognitive appraisals and perceived control. Because these psychological constructs were inferred rather than measured through validated psychometric scales, our findings capture associational patterns rather than definitive causal mechanisms. The association reported in this study links two analytically separate outputs: emotional intensity, annotated from the language of submissions, and the procedural configuration of domains, characterized from topic content and institutional structure. Perceived control and related appraisals are inferred, and procedural entrapment is offered as an interpretive framework for the association. The three annotated dimensions, polarity, intensity, and submission type, cannot distinguish anger from anxiety, resignation from indignation, or frustration directed at the process from frustration directed at the outcome. Future research should triangulate computational text analysis with self-reported surveys to validate how closely linguistic intensity corresponds to subjective psychological states, and to distinguish appraisal-driven expression from strategic intensification and platform-shaped communication conventions. Furthermore, while human–machine agreement rates for the Qwen-Turbo annotation were acceptable (κ = 0.72–0.89 across dimensions), the validation sample of 300 submissions represents less than 0.4% of the corpus. LLM-based annotation may also underperform on culturally specific forms of emotional expression common in Chinese administrative discourse, such as indirect frustration conveyed through exaggerated politeness or formally restrained language that masks strong underlying affect. These subtleties may lead to systematic underestimation of emotional intensity in certain submissions, particularly in domains where citizens adopt deferential linguistic strategies despite high levels of frustration. Second, our dataset consists of cross-sectional submissions from a single, economically developed province in China. Each record is a single submission. Accounts of prior attempts and referrals are retrospective narrative content within individual submissions; they were examined qualitatively and were not linked across records or quantified. Record linkage that traces within-citizen trajectories over time would be needed to document sustained engagement without resolution and to distinguish it from withdrawal. Findings from one platform in one province, contributed by self-selected submitters, may not generalize to other platforms, provinces, or national contexts. The specific spatial-functional misalignment observed here may vary across different regional capacities or cultural contexts with alternative expectations of governance. Third, the topic structure underlying the emotion–topic coupling analysis was derived from a single modeling configuration. The stability of topic boundaries was not formally tested across alternative random seeds, embedding models, or clustering parameters. Embedding-based representations of political-administrative text can vary with encoder choice and modeling decisions (Zhang et al., 2024), and future work could assess whether the domain-level patterns reported here replicate under alternative configurations.

Additionally, the data capture only citizens who chose to submit complaints, introducing a selection effect. Citizens who experienced procedural entrapment but ultimately disengaged—those whose trajectory more closely resembles learned helplessness through complete withdrawal from the complaint system—are absent from the dataset. Consequently, our analysis may underestimate the full psychological impact of institutional fragmentation, as it only reflects the emotional expressions of citizens who remained engaged despite procedural difficulties. Future research incorporating platform non-users or former users could help distinguish between escalation and exit as alternative responses to administrative friction.

Crucially, by focusing on collective emotional patterns, our aggregate-level analysis obscures the role of individual differences. A vital direction for future research in personality and social psychology is to examine how specific personality traits moderate individuals' susceptibility to procedural entrapment. For instance, individuals with high trait resilience or a strong baseline propensity for institutional trust may appraise bureaucratic fragmentation differently than those with high neuroticism or lower generalized trust. Future experimental or mixed-methods designs could manipulate procedural transparency and accountability cues to test how different personality profiles navigate administrative burdens. Exploring the interaction between individual personality traits and structural procedural justice could help clarify why identical administrative pathways trigger passive resignation in some citizens and active emotional escalation in others.

### 4.5 Practical implications

The findings suggest that emotional clustering within digital complaint platforms can serve as an additional source of information for governance, capturing not only dissatisfaction with outcomes but also patterns consistent with collective appraisals of procedural fairness and perceived control. High-intensity expressions appear in domains where responsibility is dispersed and procedural pathways are opaque. These emotional concentrations may therefore be interpreted as aggregated signals of procedural frustration and blocked agency, which are not immediately visible in conventional indicators such as response rates or case volumes. Emotional data offer a complementary view of administrative performance, one focused on the subjective experience of problem resolution and expectations of fairness, rather than the formal status of case handling. A simple measure—relating the share of cross-domain keywords to the breadth of domains they bridge—could offer a proxy for where coordination challenges and psychological pressure points are most likely to arise within multi-agency environments.

Seen from this angle, emotional responsiveness becomes a structural capability directed at the conditions associated with escalation. The emphasis shifts from managing expressions to examining the institutional conditions that make escalation likely: diffuse accountability, unclear procedures, and limited visibility of progress, all of which may restrict citizens' perceived influence over the administrative pathway. Emerging integrated grievance systems and AI-assisted routing can be understood as practical responses to this logic by seeking to reduce the cognitive burden and citizen-side friction created by fragmented structures. Design principles that enhance transparency, signal progress, and provide clear responsibility points may improve perceived procedural justice and reduce emotional escalation, consistent with theories of appraisal and procedural fairness. Incorporating emotional diagnostics into early-warning models could help institutions identify emerging pressure points before perceptions of unfairness accumulate into stronger collective signals, enabling proactive adjustments in coordination mechanisms and communication practices.

## 5 Conclusion

This study suggests that emotional patterns in digital complaint systems offer valuable insights into the social psychology of digitally mediated governance. Rather than merely reflecting the objective severity of a given issue, emotional escalation in these platforms appears associated with procedural entrapment—complaint contexts in which fragmented institutional pathways may reduce perceived control and diffuse accountability. Furthermore, the bridging vocabulary identified across administrative domains highlights a fundamental spatial-functional misalignment. This misalignment points to the additional cognitive and psychological demands placed on citizens when their holistic, lived experiences of localized problems are forced into segmented bureaucratic categories. From a practical and theoretical standpoint, negative emotional expressions should not be dismissed merely as individual-level noise or administrative friction. Instead, they can serve as collective diagnostic signals, pointing to where institutional design may be associated with diminished perceived agency. By conceptualizing emotional responsiveness as the capacity to detect and address such bottlenecks, public organizations can move beyond basic procedural compliance. In this sense, the study aligns with the United Nations' Sustainable Development Goal 16 (Peace, Justice and Strong Institutions) by suggesting that the development of more effective and accountable institutions may also depend on how administrative pathways are psychologically experienced by citizens.

## Data Availability

The original contributions presented in the study are included in the article/Supplementary material, further inquiries can be directed to the corresponding author.
