# Supplementary material for: Trapped in the process: procedural justice and emotional escalation in digital citizen-state interactions
Source: Front Psychol. 2026 Jul 29;17:1844065. doi: 10.3389/fpsyg.2026.1844065 (PMC13461590; doi:10.3389/fpsyg.2026.1844065)
Supplement: Supplementary file 1 [file Data_Sheet_1.pdf]

Supplementary Material

1 SUPPLEMENTARY TABLES AND FIGURES

1.1 Figures

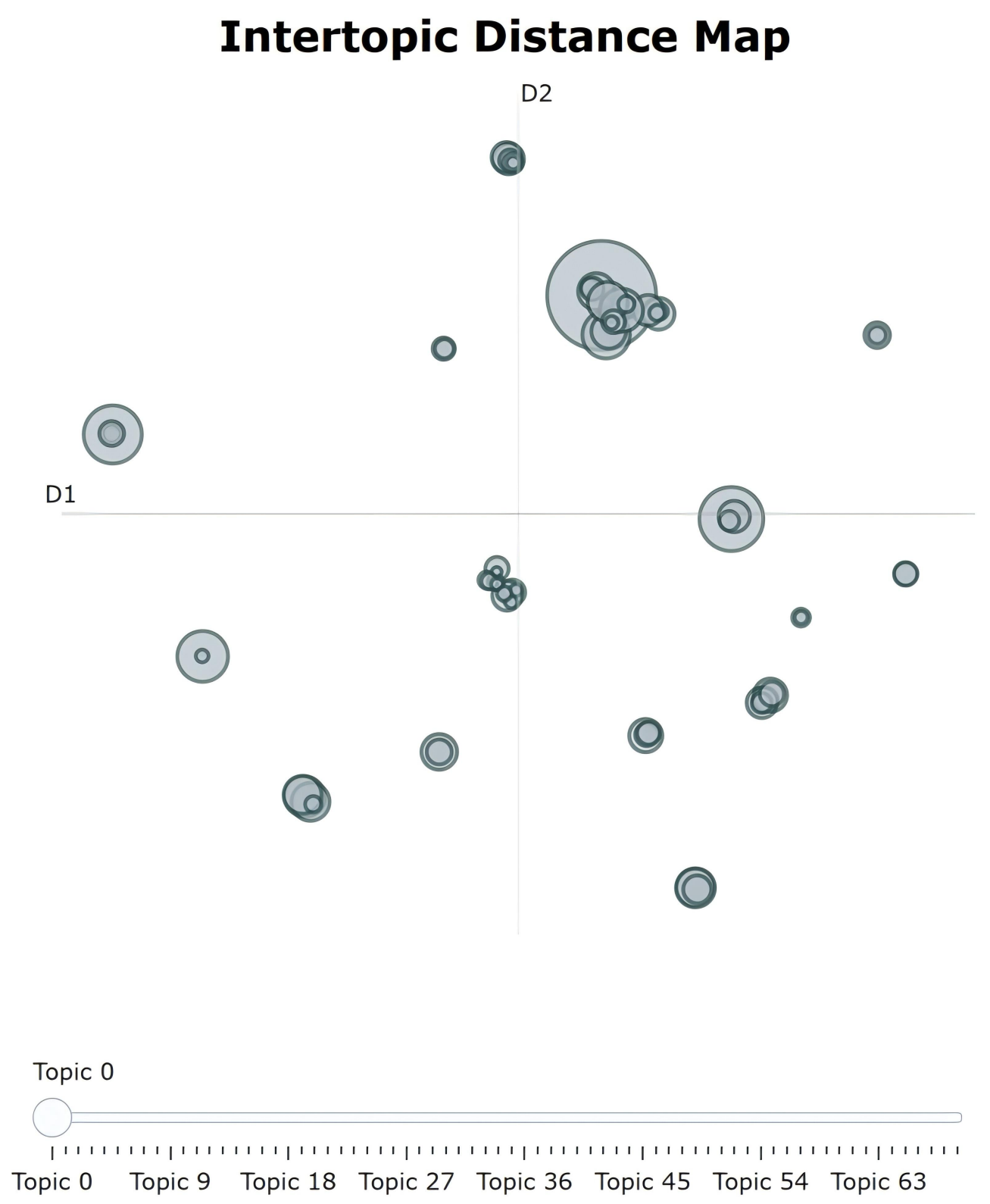

Figure S1. Two-Dimensional Projection of Initial Topic Distribution (70 Topics).

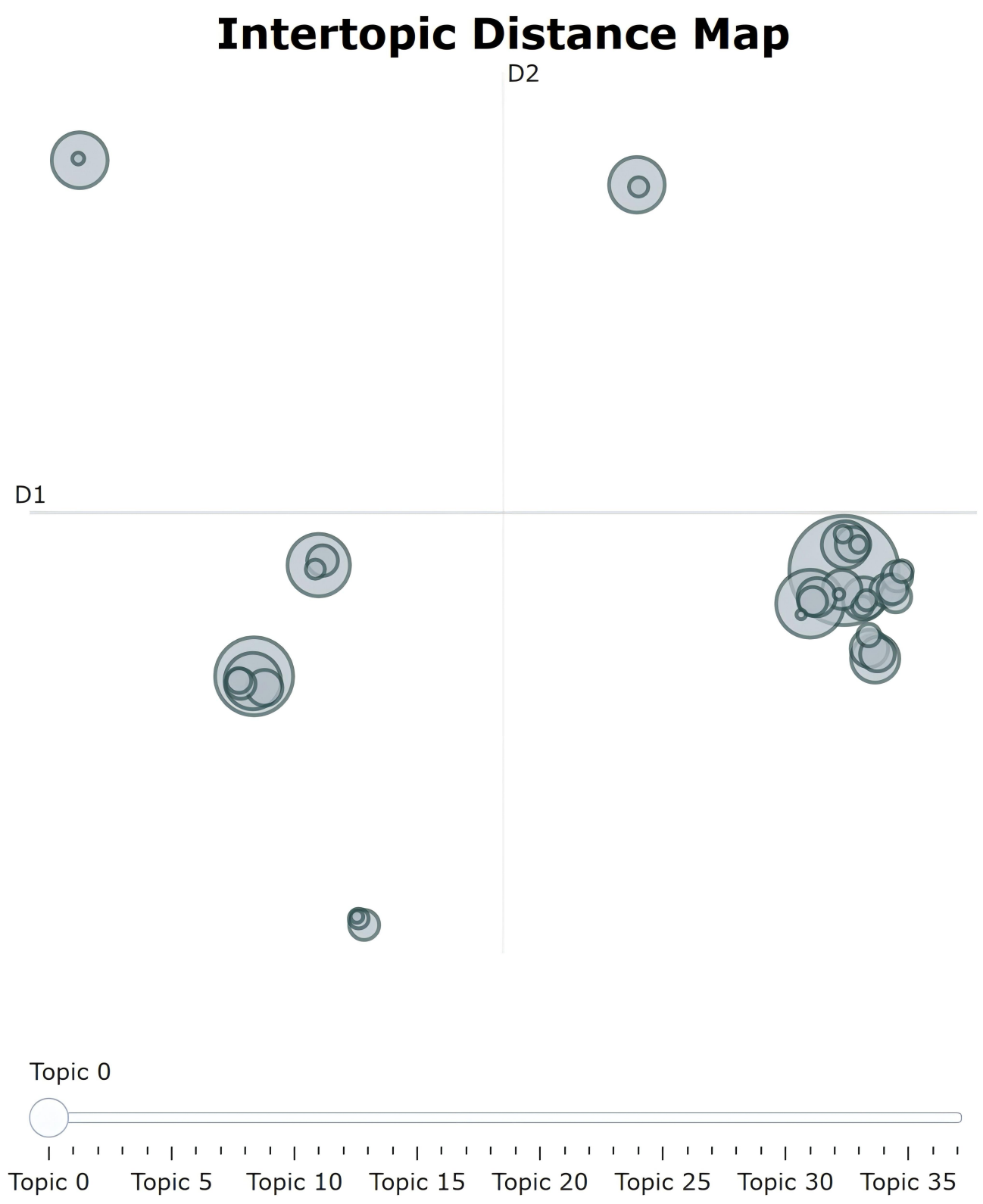

Figure S2. Two-Dimensional Projection of Refined Topic Distribution (38 Topics).

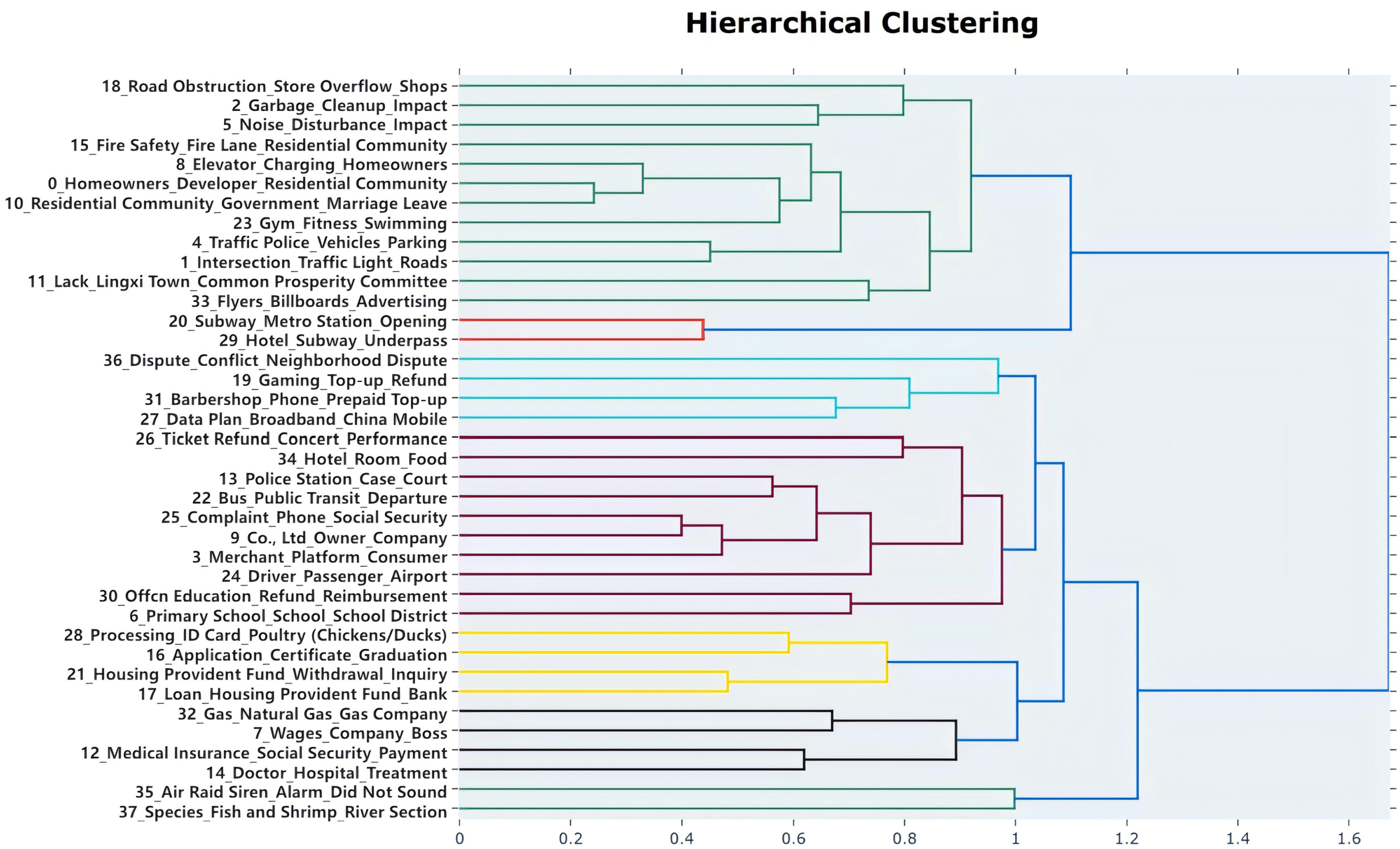

Figure S3. Hierarchical Clustering Dendrogram of Second-Level Topics .

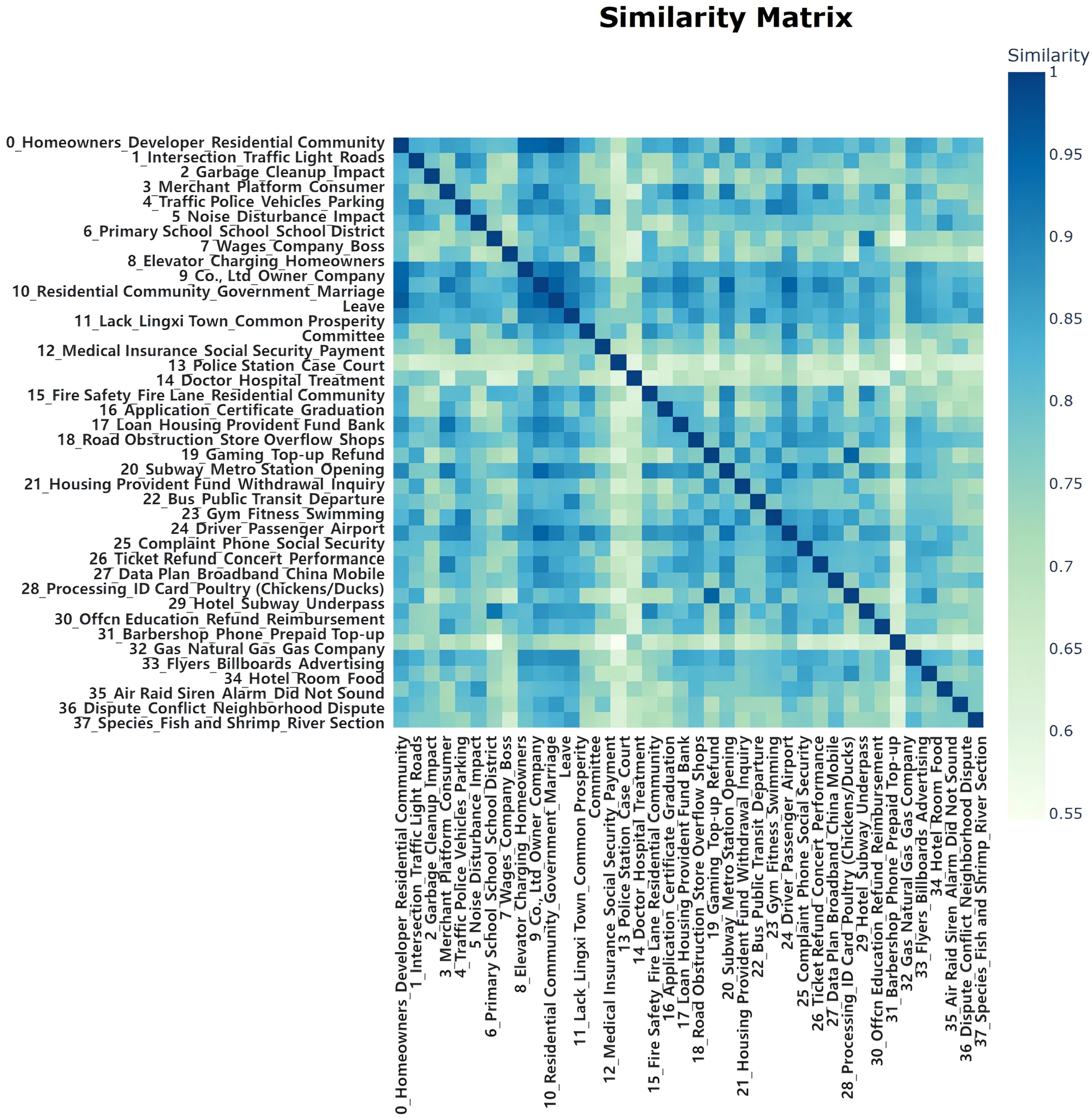

Figure S4. Pairwise Topic Similarity Matrix .
